# Activity Components from *Gynostemma pentaphyllum* for Preventing Hepatic Fibrosis and of Its Molecular Targets by Network Pharmacology Approach

**DOI:** 10.3390/molecules26103006

**Published:** 2021-05-18

**Authors:** Yumeng Zhang, Guohui Shi, Zhonghua Luo, Jiewen Wang, Shao Wu, Xiaoshu Zhang, Yuqing Zhao

**Affiliations:** 1 School of Functional Food and Wine, Shenyang Pharmaceutical University, Shenyang 110016, China; yumeng_1229@163.com (Y.Z.); zym122908@126.com (G.S.); li1sheng1115@163.com (Z.L.); li1sheng1113@163.com (J.W.); li1sheng1314@163.com (S.W.); 2 Key Laboratory of Structure-Based Drug Design & Discovery, Ministry of Education, Shenyang Pharmaceutical University, Shenyang 110016, China

**Keywords:** *G. pentaphyllum*, hepatic fibrosis, network pharmacology, PI3K/Akt signaling pathway

## Abstract

Hepatic fibrosis would develop into cirrhosis or cancer without treating. Hence, it is necessary to study the mechanism and prevention methods for hepatic fibrosis. *Gynostemma pentaphyllum* is a traditional medicinal material with a high medicinal and health value. In this study, nineteen compounds obtained from *G. pentaphyllum* were qualitative and quantitative by HPLC-FT-ICR MS and HPLC-UV, respectively. Among them, the total content of 19 gypenosides accurately quantified reaches 72.21 mg/g and their anti-proliferation against t-HSC/Cl-6 cells indicated compound **19** performed better activity (IC50: 28.1 ± 2.0 μM) than the other compounds. Further network pharmacology study demonstrated that compound **19** mainly plays an anti-fibrosis role by regulating the EGFR signaling pathway, and the PI3K–Akt signaling pathway. Overall, the verification result indicated that compound **19** appeared to be nontoxic to LO2, was able to modulate the PI3K/Akt signal, led to subG1 cells cycle arrest and the activation of mitochondrial-mediated apoptosis of t-HSC/Cl-6 cells for anti-hepatic fibrosis.

## 1. Introduction

Hepatic fibrosis was a public health problem that could cause severe morbidity and mortality. Hepatic fibrosis was an excessively traumatic repair caused by chronic liver disease [1]. Repeated or continuous hepatitis or necrosis could lead to the formation of liver fibrosis, and further cause portal hypertension, liver failure, and even death [2,3]. However, there was currently no ideal treatment.

As homology of medicine and food, *Gynostemma pentaphyllum* (Thunb.) Makino (*G. pentaphyllum*), named “Jiaogulan” and “Southern ginseng”, which was a perennial liana herb, is most distributed in China, Japan, Korea, etc. Many recent reports have shown that *G. pentaphyllum* exhibits varieties of biological activities, such as anti-tumor [4,5], anti-inflammation [6,7], anti-oxidative [8], immunity improvement [9], treatment of chronic bronchitis and gastritis [10,11], treatment of type 2 diabetes [12,13,14,15], reducing the risk of cardiovascular diseases [16], and liver protection [17,18]. In 1986, *G. pentaphyllum* was listed as the most “precious Chinese medicinal herb” to be developed in the “China spark program”, which was sponsored by the State Science and Technology Commission. On 5 March 2002, *G. pentaphyllum* was added to the list of functional foods by the Ministry of Public Health of China [19]. Currently, there were more than 80 kinds of medicines, and health products, using *G. pentaphyllum* as raw materials.

Utilizing various techniques, such as omics, high-throughput screening, network visualization, and network analysis can help us better understand the molecular mechanism of diseases and the pharmacological mechanism of drugs from a multidimensional perspective [20,21]. Network pharmacology was an accepted way for the visualization and construction of the drug–targets–disease framework, which could help us understand the correlation between drugs and targets effectively.

The phytochemical investigations of *G. pentaphyllum* revealed that the main bioactive constituents are a series of dammarane-type triterpene saponins named gypenosides, which are structurally similar to ginsenosides [22]. In the previous study, the flash homogenate extraction method, which was a convenient, fast, and efficient extraction process that can effectively shorten room temperature extractions and retain more effective ingredients in the extracted plant [23], was used to extract the total gypenoside from *G. pentaphyllum*. Moreover, nineteen individual gypenosides were isolated from *G. pentaphyllum*. However, there has been no in-depth research on the mechanism of gypenoside fully established. In the present study, qualitative analysis of nineteen individual gypenosides was explored by HPLC-FT-ICR MS, and the contents of the gypenosides in the extracts were tested by HPLC. In addition, the anti-proliferation of the individual gypenosides was investigated for a comparative purpose, and this is the first time to investigate the potential mechanisms of the most active compound on the basis of network pharmacology, based on a previous study [18].

## 2. Results and Discussion

### 2.1. Chemical Studies of Gypenosides Extracted from G. pentaphyllum

**Figure 1:**
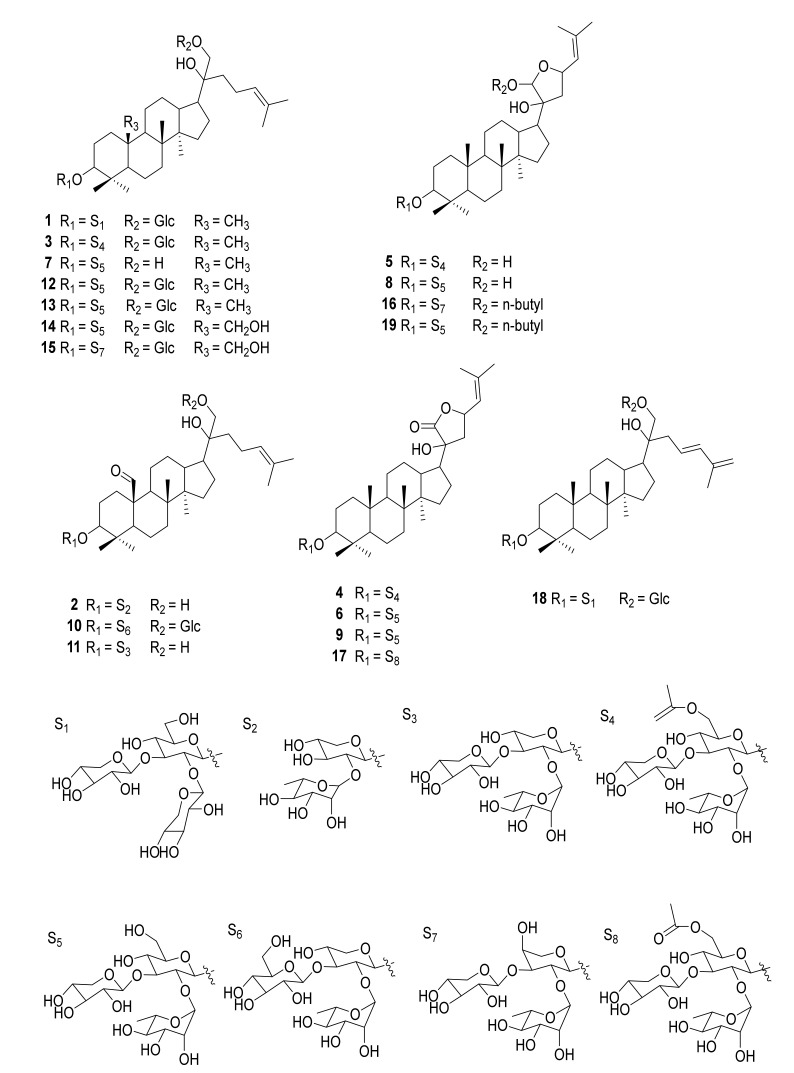
The structures of compounds **1–19**.

**Figure 2:**
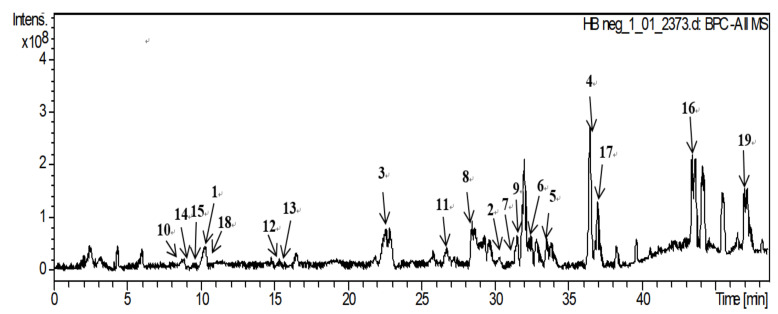
The HPLC-FT-ICR MS chromatogram of *G. pentaphyllum*.

To explore whether the nineteen individual gypenosides (Figure 1) isolated from *G. pentaphyllum* were artifacts, the compounds were analyzed by the HPLC-FT-ICR MS method for qualitative analysis. Gradient elution was used to increase the resolution of the HPLC analysis because of the complexity of the components contained in *G. pentaphyllum*. After comparing acetonitrile–water and methanol–water as mobile phases, we found that the separation effect of the acetonitrile–water system was better. For determination of the conditions for MS analysis, we tested both positive and negative ionization modes. The results showed that the negative ionization mode could provide intense proton signals, [M−H]^−^. The peak information of HPLC-FT-ICR MS analysis is provided in Table 1, and the HPLC-FT-ICR MS chromatograms of *G. pentaphullum* along with the HPLC-FT-ICR MS spectra are shown in Figure 2. The determination of the characteristics of the 19 tested compounds was carried out by referring to both the HPLC information of the mixed stock solution and the information of the mass spectrum. Based on this information, the peak information was finally determined. This study established a rapid detection method for saponins in *G.* *pentaphyllum* for the first time.

**Figure 3:**
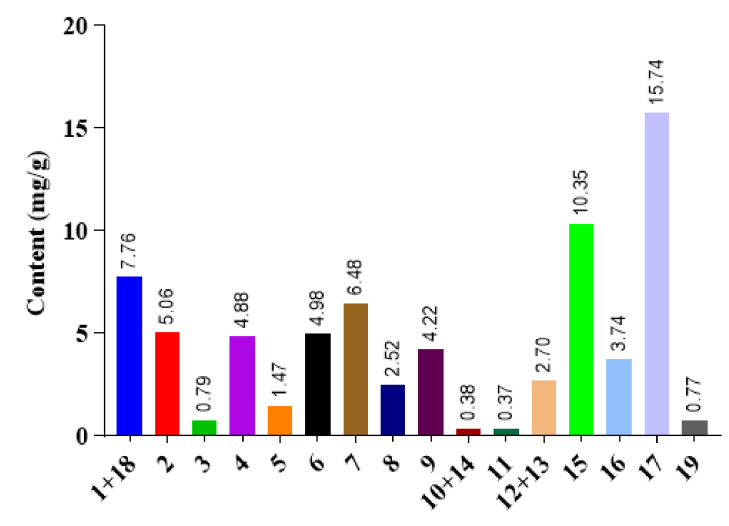
The content of compounds in *G. pentaphyllum* extracts.

The results of linearity, precision, accuracy and stability experiments are presented in Table 2. According to the results, the method was proved to be precise, stable and reliable for analysis. The quantitative analyses were performed by external standard methods. Nineteen individual gypenosides were obtained in all the experimental extracts. It was found that the total content of the 19 compounds was 72.21 mg/g. Among them, compound **17** had the highest content with 15.74 mg/g, followed by compound **15** with 10.35 mg/g. In contrast, compound **11** owned the lowest content at only 0.37 mg/g (Figure 3).

### 2.2. Specific Anti-Proliferative Effect of Individual Gypenosides on t-HSC/Cl-6 Cells

Whether drugs or nutritional supplement, it is essential to reduce the side effects on the human body as much as possible [24]. Thus, LO2 cells were selected to evaluate the specificity of compounds to t-HSC/Cl-6 cells in the study. As shown in Table 3, all the compounds showed no cytotoxicity.

In addition, the MTT results of the anti-proliferative effects against the t-HSC/Cl-6 cells activity indicated that compound **19** performed effective anti-proliferative effects against t-HSC/Cl-6 with an IC50 value of 28.1 ± 2.0 µM. It was speculated that compound **19** displaying the strongest inhibitory effect may be related to the synthesis of a five-membered unsaturated ketone ring in the side chain ring and the n-butyl at the C-21 position.

### 2.3. Network Pharmacology Analysis

#### 2.3.1. Screening of Common Targets and Constructing of “Protein–Protein Interaction” (PPI) Networks between Gypenoside and Liver Fibrosis

**Figure 4:**
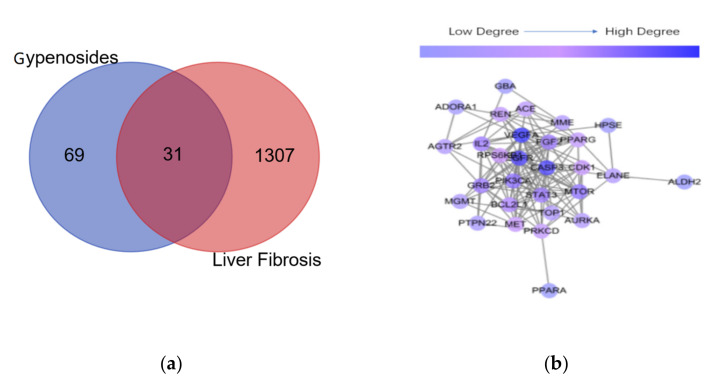
Drug–disease interaction network analysis. (**a**) The Venn diagram of targets; (**b**) the PPI network analysis of gypenosides and liver fibrosis. In a, the overlapping genes were selected as key targets for further analysis.

As shown in Figure 4a, 69 targets of compound **19** reported were retrieved from the Swiss target prediction database and 1307 anti-liver fibrosis targets were obtained after high-possibility screening in the GeneCards database. Afterwards, we selected 31 targets as intersection targets for further study. The common targets of gypenoside and liver fibrosis were imported into the STRING database for analysis. There were 31 nodes and 136 edges in the network, with the average node degree value being 8.77 (Figure 4b). The higher degree value indicated that it may be the interaction between the component target and the disease target, and the node plays an important role in the network. Subsequently, the targets that are larger than the average node degree value were arranged in the order of VEGFA, EGFR, CASP3, PIK3CA, MTOR, FGF2, GRB2, STAT3, BCL2L1, RPS6KB1, CDK1.

#### 2.3.2. KEGG Pathways Analysis and GO Enrichment

**Figure 5:**
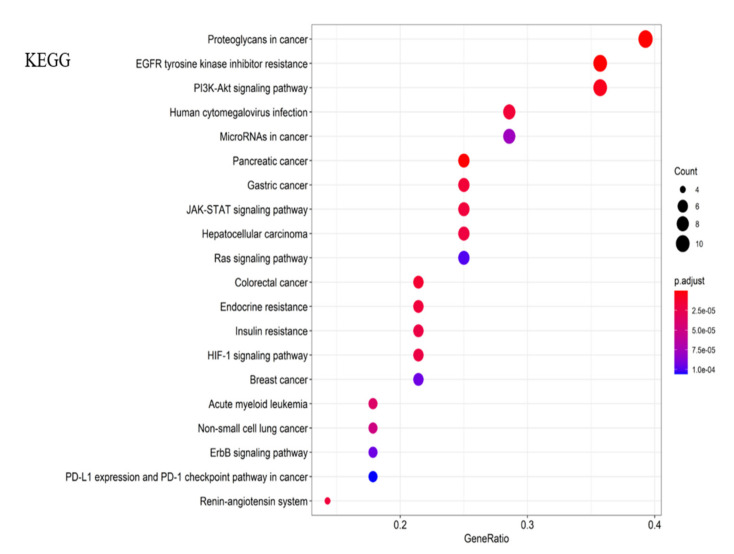
Histogram of the KEGG pathway of gypenosides.

**Figure 6:**
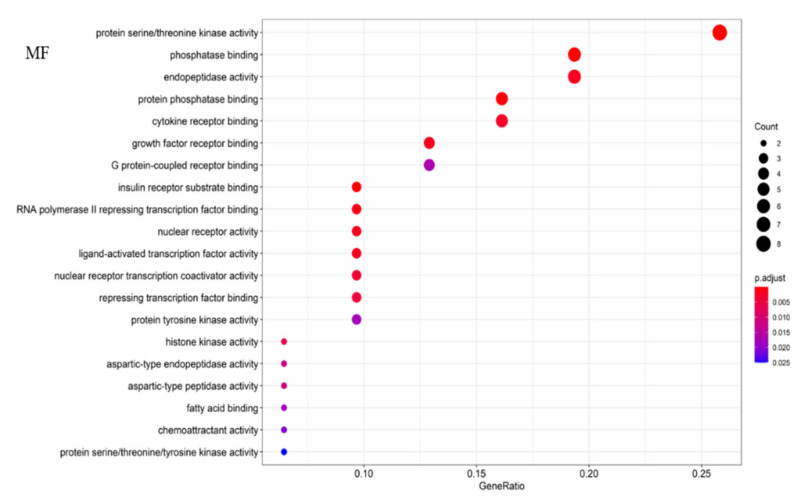

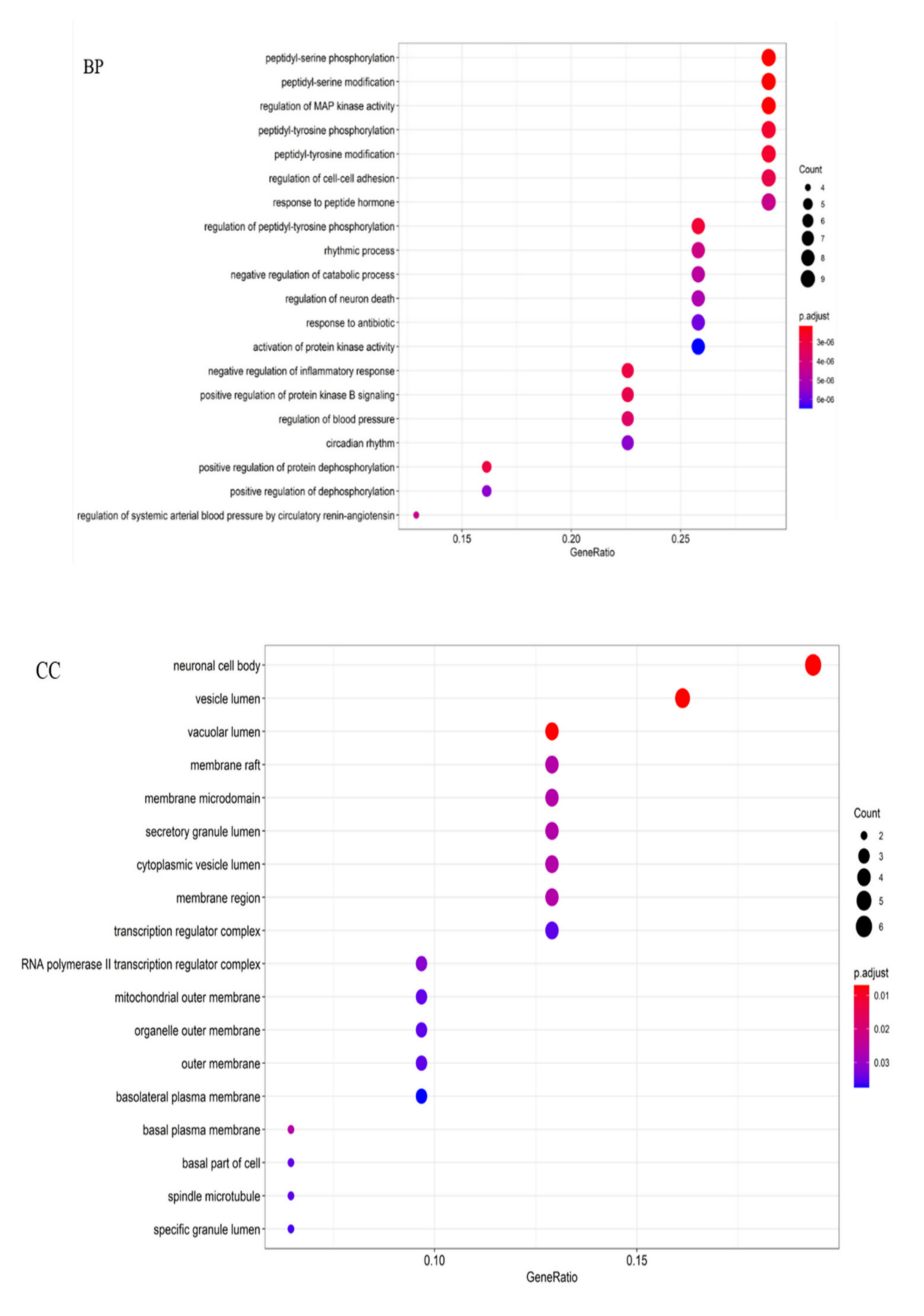
GO analysis bubble chart of gypenosides. **MF**: molecular function; **BP**: biological process; **CC**: cellular component.

**Figure 7:**
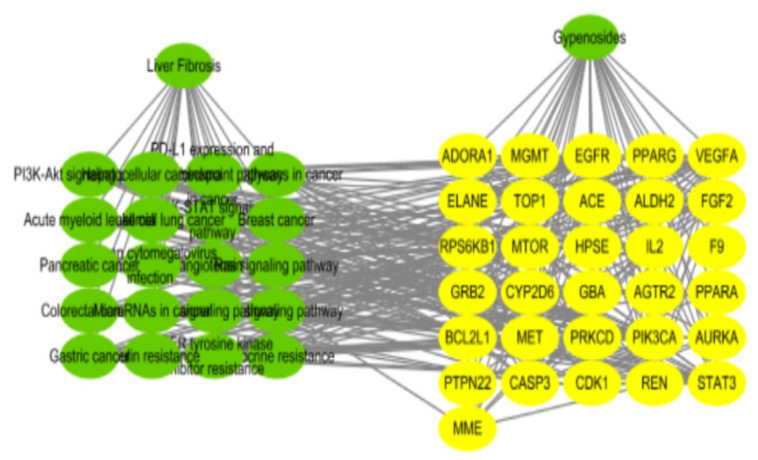
Interactive network of “gypenosides–target–pathway–liver fibrosis”.

The KEGG pathways analysis was conducted for the PPI network, and the results in Figure 5 showed that gypenoside played an anti-liver fibrosis effect through EGFR tyrosine kinase inhibitor resistance, proteoglycans in cancer, pancreatic cancer, the PI3K–Akt signaling pathway, etc. The top 20 results of GO enrichment were displayed in Figure 6, which included biological processes (BP), molecular functions (MF), and cellular components (CC). Overall, the “component–target–pathway–disease” pathway diagram was shown in Figure 7. There were 53 nodes and 317 edges in the network, of which 31 yellow circular nodes were common targets, and 19 green circular nodes were the selected KEGG pathway. The degree values of the common targets are EGFR, PIK3CA, MTOR, GRB2, VEGFA, RPS6KB1, STAT3, CASP3, and FGF2 in descending order, and the degree values of the pathways in descending order are proteoglycans in cancer, PI3K–Akt signaling pathway, EGFR tyrosine kinase inhibitor resistance, microRNAs in cancer, human cytomegalovirus infection, gastric cancer, and hepatocellular carcinoma. It can be seen that gypenoside has a wide range of pharmacological effects. In addition to its excellent anti-liver fibrosis effect, it also has significant effects on gastric cancer and lung cancer.

### 2.4. The Anti-Hepatic Fibrosis Mechanism of Compound ***19***

**Figure 8:**
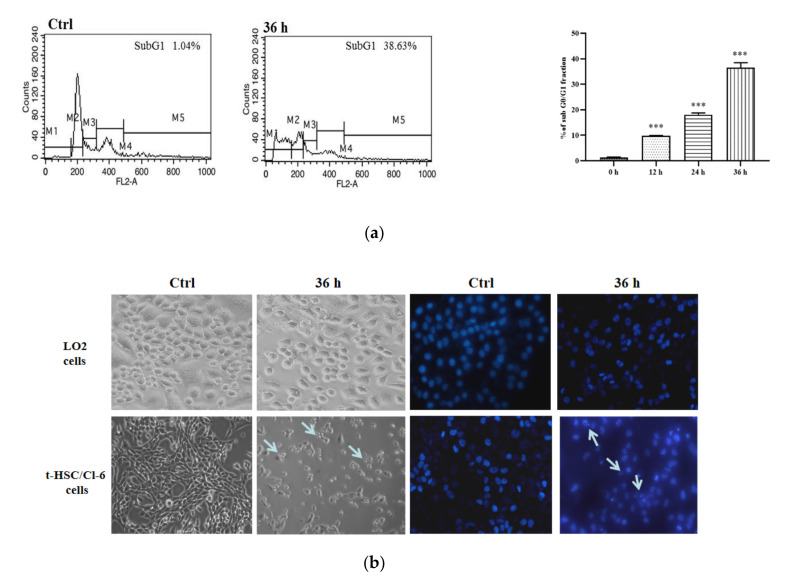
(**a**) The cell cycle analysis by flow cytometry of compound 19. *** *p* < 0.001; (**b**) morphological changes and the nuclear morphological changes of t-HSC/Cl-6 cells and LO2 cells treated with compound **19** for 36 h. Control cells were incubated with DMSO. The arrow indicates apoptotic cells.

HSC activation was vital for the formation of liver fibrosis as it is the main source of activated myofibroblasts that produce ECM in the liver. Due to the excellent anti-proliferative activity of compound **19**, the flow cytometric analysis was used to explore whether compound **19** affected the t-HSC/C1-6 cells cycle progression. As shown in Figure 8a, the cells in the subG1 phase increased to 38.63% after being treated with compound **19** for 36 h compared to 1.04% in the control group, which indicated that compound **19** could prevent cell division by blocking DNA replication. Subsequently, Western blotting was used to detect the effect of compound **19** on cell cycle regulatory proteins. Compound **19** decreased cyclin D1 expression and caused cell arrest in the subG1 phase. The subG1 phase of the cell cycle can be blocked by downregulating the expression of cyclin D1 [25].

It was the first time to explore whether compound **19** could cause apoptosis in t-HSC/C1-6 cells and LO2 cells. As displayed in Figure 8, apoptotic bodies and cell shrinkage were observed in treated t-HSC/Cl-6 cells after 36 h, which indicated apoptosis. On the contrary, LO2 cells were well alive, and no shrinkage was seen. In additional, DAPI staining was used to determine the morphological changes of the nuclei in the cells. Typical apoptotic nuclear changes were observed, including fragmented apoptotic bodies, and condensation and shrinkage of nuclei. However, compound **19** did not appear to have any specific effect on the nucleus of the LO2 cells. It was confirmed that compound **19** was specific for t-HSC/Cl-6 cells.

**Figure 9:**
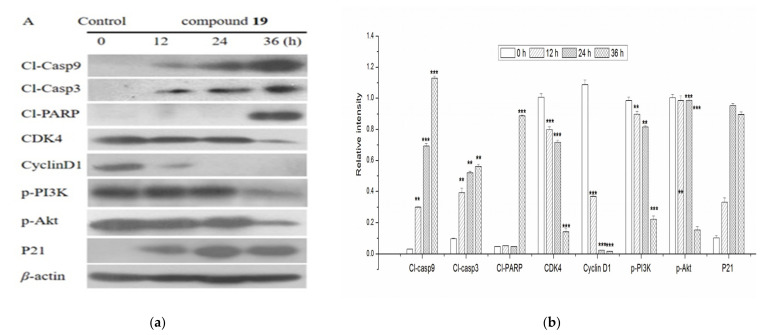
(**a**) The mechanism of compound **19** inducing apoptosis and arresting t-HSC/Cl-6 cells cycle; (**b**) effects of compound **19** on the ratio of protein in t-HSC/Cl-6 cells. Data are presented as the means ± SD of three independent experiments. ** *p* < 0.01, *** *p* < 0.001 compared with *β*-actin.

**Figure 10:**
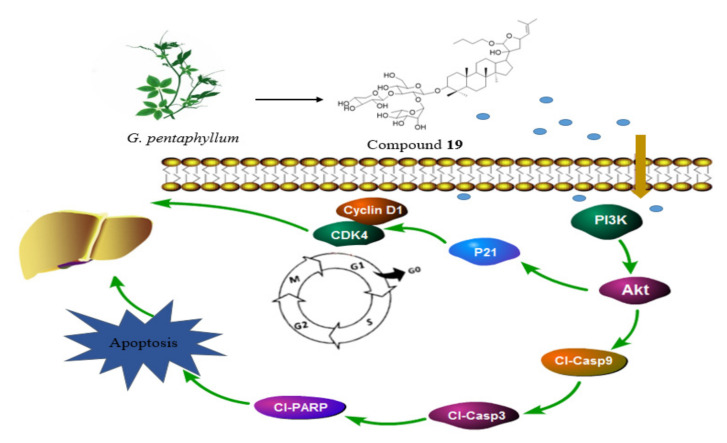
Possible anti-mechanisms of compound **19** in t-HSC/Cl-6 cells.

To confirm that compound **19** induces possible activation of CDKs in addition to the activation of cyclins, the expressions of CDK4 were also evaluated. P21 was critical in the progression of the cell cycle and was associated with liver fibrosis [26]. Compound **19** caused significant accumulation of p21 and inhibition of CDK4 (Figure 5). As displayed in Figure 9, compound **19** inhibited the expression of the phosphorylated-PI3K and -Akt proteins as time increased. The research indicated that the activation of the PI3K/Akt signaling pathway played a central role in liver fibrosis, and certain therapeutic drugs target this pathway to exert an anti-liver fibrosis effect [27,28]. Noteworthy, the PI3K/Akt signaling pathway plays an important role in promoting cell proliferation and inhibiting apoptosis [29]. Wang et al. indicated that microRNA-29b can prevent the formation of liver fibrosis by inhibiting the PI3K/Akt pathway to induce HSC apoptosis (Wang et al., 2014). It was well known that activated caspase was the biochemical marker for cell apoptosis [30]. The caspase family of cysteine proteases acts as a pivotal role in the occurrence and development of apoptosis. Figure 9 shows that the expression levels of cleaved caspase-9 and cleaved caspase-3 were activated with in a dose-dependent manner, after being treated with compound **19**. These results suggested that compound **19** exerted anti-growth activity against t-HSC/Cl-6 cells through a caspase-dependent pathway (see Figure 9). To summarise, the possible anti-mechanisms of compound **19** in t-HSC/Cl-6 cells as shown in Figure 10.

## 3. Materials and Methods

### 3.1. Materials and Regents

The standards of compounds **1** to **19** (>98.0%) were isolated from *G. Pentaphyllum* in our previous study [18]. The flash extractor was obtained by All Herbal Scientech, LLC (JHBE-20A, Beijing, China). Acetonitrile and methanol of HPLC grade were obtained from Fisher Scientific (Pittsburgh, PA, USA). Purified water was purchased from Wahaha (Hangzhou, China). All other chemical agents were of analytical grade, bought from Qingdao Haiyang Chemical Co. Ltd. (Shandong, China). MTT reagent was acquired from Sigma-Aldrich (St. Louis. MO, USA). DMSO was purchased from Sigma Chemical Co. (St. Louis, MO, USA). DMEM medium and FBS were bought from Thermo Fisher Scientific Co., Ltd. Cl-caspase-3, Cl-caspase-9, Cl-PARP (Cell Signaling Technology, Inc., Danvers, MA, USA), Cyclin D1, CDK4, P21 (Proteintech Group, Inc., Wuhan, China), *β*-actin (Nanjing Jiancheng Bioengineering Institute, Nanjing, China), and Phosphatidylinositol-3 kinase (PI3K), phosphorylated-Akt were obtained from Cell Signaling Technology, Inc (Danvers, MA, USA).

### 3.2. Extraction Procedures

The samples of *G. pentaphyllum* were purchased from Bozhou Yonggang Co., Ltd. (Anhui, China) and identified as the aerial part of *G. pentaphyllum* by Prof. Jincai Lu of Shenyang Pharmaceutical University, and its voucher specimen was deposited in Museum of Traditional Chinese Medicine, Shenyang Pharmaceutical University (no. 18066). After collecting, the 73% ethanol-to-*G. pentaphyllum* which dried at 40 °C for 3 days in a ratio of 41:1 mL/g were mixed and placed in the flash extractor for 6.5 min, which was optimized using response surface optimization in the previous study [18].

### 3.3. HPLC-FT-ICR MS Analysis

The HPLC-FT-ICR MS was carried out on an Agilent 1260 system with an FT-ICR MS-equipped electrospray ionization (ESI) interface and a 7.0 T superconducting magnet. The HPLC mobile phase consisted of water (A) and acetonitrile (B) at 0.8 mL/min flow through an Agilent 5 HC-C18 column (250 × 4.6 mm). Mobile phase elution system was performed in linear gradient (34–38% B at 0–15 min, 38–40% B at 15–20 min, 40–47% B at 20–25 min, 47–60% B at 25–35 min, 60–80% B at 35–40 min, 80% B at 40–50 min). The column temperature was maintained at 25 °C. The injection volume was 20 μL while the UV detection monitored at 203 nm.

The ESI source conditions were as follows: a nebulizer gas pressure of 4.0 bar, a dry gas flow rate of 8.0 mL/min, a capillary voltage of 3.5 kV, end plate offset of 500 V and a transfer capillary temperature of 200 °C. Full-scan MS data was acquired over an m/z range of 150–2000, and the collision energy was initially set at 10 eV for the MS experiments of the preferred ions and then modified according to the fragments. In the full-scan mass spectra, most of the constituents exhibited [M − H]^−^ in negative ion mode under the soft ESI condition. Precursor ions were subjected to collision-induced dissociation (CID) to generate the fragment ions, and the fragmentation patterns were proposed for the structural identification of constituents. The FT-ICR mass spectrometer was calibrated in negative mode according to the manufacturer’s instructions using a solution of NaTFA (0.05–0.1 mg/mL in 50:50 ACN:H2O). FT-MS control, Bruker Compass–Hystar and data analysis software (Bruker Daltonics, Bremen, Germany) were used to control the equipment and for data acquisition and analysis.

### 3.4. Simultaneous Determination of Individual Gypenosides

Analyses were performed on a CXTH-3000 HPLC system (Beijing Chuangxintongheng Science and Technology Co., Ltd. Beijing, China). The measurement conditions were consistent with Section 2.3. The 1 mg/mL stock solutions were diluted and used for building the linearity curve. Each linearity curve consisted of five different concentrations and tested in triplicate. Intra- and inter-day variations of standard and sample solutions which had certain concentrations were tested. The standard solution was injected six times within 1 day to test intra-day variation and the RSD were calculated. Six individual sample solutions were injected for three consecutive days to test the inter-day variation and the values of RSD were calculated. The recovery test was performed to evaluate the accuracy of the method. The stability experiment was injecting the same sample solutions in 0, 0.5, 1, 2, 4, 6, 8, 12 h at 25 °C and the results were expressed by RSD value.

### 3.5. Cell Proliferation Assays

The cell viability was assessed with the MTT assay. The human hepatic stellate cells (t-HSC/Cl-6) and human normal liver cells (LO2) bought from the Cell Bank of Chinese Academy of Sciences (Shanghai, China) were seeded in 96-well plates for 24 h and cultured with high glucose including 10% fetal bovine serum, and 1% penicillin/streptomycin were used to culture cells at 37 °C under 5% CO_2_ and then were treated with serial dilutions of the compounds (25, 50, 100, 200, 400 μM) for another 36 h. For MTT assay, ten microliters of MTT reagent (5 μg/mL) was added to each well after removing all the liquid in the wells, and incubated at 37 °C in a humidified atmosphere of 5% CO_2_ air for 4 h. At the end of incubation, the growth medium was removed and replaced with 100 μL of DMSO at room temperature and agitating on a vortex for 20 min. The absorbance was determined at 490 nm as reference on a Bio-Rad micro-plate reader.

### 3.6. Network Pharmacology

#### 3.6.1. Screening of Common Targets of Compound 19 and Liver Fibrosis

The structure of compound **19** was submitted to the Swiss target prediction database (STP, http://www.swisstargetprediction.ch/, accessed on 19 October 2020) to determine the target of gypenoside. The GeneCards database (http://www.genecards.org/, accessed on 19 October 2020) was used to find targets related to liver fibrosis, and the screening condition was set as “score > 10”. Then, the screened targets were mapped to obtain the intersection target of gypenoside and liver fibrosis.

#### 3.6.2. The Enrichment Analysis of Gene Ontology (GO) and KEGG Pathway

Metascape (https://metascape.org/gp/index.html#/main/step1, accessed on 19 October 2020) was applied to evaluate the KEGG signaling pathway and GO enrichment to clarify the function of the targets and their function in signal transduction.

#### 3.6.3. Construction of “Component–Target–Pathway–Disease” Network

The targets of gypenoside on liver fibrosis were submitted to STRING (https://string-db.org/cgi/input.pl, accessed on 19 October 2020) and then the network was visualized by Cytoscape 3.6.0.

### 3.7. Cell Cycle Test

The cell cycle analysis was performed using a previous method [31]. The t-HSC/C1-6 cells were cultured in the presence of 50 μM compound **19** and DMSO for 12 h, 24 h, and 36 h. Briefly, treated and untreated cells were trypsinized, harvested, and washed with PBS. Then the cells were fixed with 75% ethanol overnight at 20 °C. Fixed cells were washed with PBS and stained with propidium iodide solution in the dark for 4 h. Cell cycle distribution was analyzed using FACScan (Becton Dickinson Immunocytometry Systems, San Jose, CA, USA).

### 3.8. Cell Apoptosis Analysis

For cell morphology analysis, t-HSC/C1-6 cells and LO2 cells were grown in 6-well plates. After 24 h, compound **19** was incubated with the cells for another 36 h and 0.1% DMSO was used to culture control cells. The cellular morphology was observed with phase contrast microscopy (Olympus, Tokyo, Japan). Then the cells were rinsed with PBS and resuspended in 1 mL of 0.1% DAPI prepared in methanol for 15 min. PBS was used to wash the cells three times. Nuclear morphology was observed by a fluorescent microscope (Olympus BH-2, Osaka, Japan).

### 3.9. Western Blotting Test

RIPA lysis buffer was used to extract total cellular proteins, and the protein concentration was determined by bicinchoninic acid. Protein samples (20 μg/lane) were loaded onto 10% SDS–PAGE gels for electrophoresis and then the transmembrane was conducted. After that, blocking of polyvinylidene fluoride (PVDF) membrane was carried out using 5% bovine serum albumin in Tris-buffered saline Tween-20 (TBST) buffer for 1 h, subsequently probing with a primary antibody (1:1000 dilution), such as p-PI3K, p-Akt, Cl-caspase-3, Cl-caspase-9, Cl-PARP, CDK4, Cyclin D1, or P21, at 4 °C overnight. Then, TBTS was used to wash the membrane. The PVDF membranes were incubated with the secondary antibody (1:5000 dilution) for 2 h. X-ray films were employed to develop the signals [32].

### 3.10. Statistical Analysis

All data represent the mean ± S.D. of three independent experiments. Analysis of variance was performed by two-way ANOVA procedures, using Prism 5.0 (GraphPad Software Inc., San Diego, CA, USA). Western blot band intensity was analyzed using ImageJ and statistical analyses were conducted using SPSS software package (version 16.0, IBM Corp., Armonk, NY, USA).

## 4. Conclusions

The study provided a quick and accurate method for the identification and quantification of gypenosides extracted from *G. pentaphyllum*. Moreover, further activity screening proved that compound **19** with an IC50 of 28.1 ± 2.0 μM performed better liver fibrosis activity than other compounds. The network relationship of compound **19** against liver fibrosis was established for the first time with the help of network pharmacology, and it was verified that compound **19** was able to modulate the PI3K/Akt signal and then induce subG1 arrest and activate mitochondrial-mediated apoptosis of t-HSC/Cl-6 cells for preventing liver fibrosis. *G. pentaphyllum* can be used as an anti-hepatic fibrosis plant, and compound **19** may represent attractive ingredients.

## Figures and Tables

**Table 1:** The peak information of HPLC-FT-ICR MS analysis.

No.	Retention Time	[M−H]^+^	Error	Compounds
1	8.37	1075.57105	−1.49	**10**
2	9.19	1077.58650	−1.30	**14**
3	9.90	1047.57184	2.57	**15**
4	10.09	1047.56959	4.72	**1**
5	10.19	1045.55885	0.03	**18**
6	15.21	1061.59252	−2.20	**12**
7	15.30	1061.59273	−2.39	**13**
8	23.39	1103.60267	−1.74	**3**
9	26.67	883.50701	−1.08	**11**
10	28.49	913.51883	−2.42	**8**
11	30.38	751.46499	−1.59	**2**
12	31.38	899.53926	−2.11	**7**
13	31.76	911.50313	−2.36	**9**
14	32.51	911.50094	0.04	**6**
15	33.13	955.52763	−0.46	**5**
16	36.41	953.51526	−3.87	**4**
17	36.92	953.51415	−2.73	**17**
18	43.40	953.55206	−4.33	**16**
19	47.01	969.58297	−3.87	**19**

**Table 2:** The methodology investigation results of simultaneous determination of **19** gypenosides.

Compounds	Linear Regression Data			Precision RSD (%)		Stability RSD (%)
	Linearity Curve	R2	Linear Range (mg/mL)	Intra-Day	Inter-Day	
**1**	Y = 4 × 106x + 2.764 × 104	0.9992	0.01–1	1.86	1.38	2.47
**2**	Y = 1 × 106x + 8.823 × 103	0.9994	0.01–1	2.08	2.57	2.20
**3**	Y = 5 × 106x + 1.406 × 104	0.9993	0.01–1	1.57	2.02	1.93
**4**	Y = 5 × 106x + 2.068 × 104	0.9991	0.01–1	1.62	1.54	1.52
**5**	Y = 1 × 106x + 8.790 × 103	0.9994	0.01–1	1.05	1.99	1.76
**6**	Y = 3 × 106x + 1.062 × 104	0.9991	0.01–1	1.13	1.07	1.35
**7**	Y = 4 × 106x + 1.333 × 104	0.9991	0.01–1	1.25	0.98	1.88
**8**	Y = 4 × 106x + 2.874 × 103	0.9991	0.01–1	1.37	2.88	1.06
**9**	Y = 6 × 106x + 1.479 × 104	0.9989	0.01–1	1.67	1.49	2.50
**10**	Y = 5 × 106x − 7.298 × 103	0.9992	0.01–1	1.27	2.38	2.04
**11**	Y = 7 × 106x + 4.892 × 104	0.9992	0.01–1	1.94	1.94	1.69
**12**	Y = 5 × 106x + 4.022 × 104	0.9992	0.01–1	2.11	1.74	2.51
**13**	Y = 3 × 106x + 2.574 × 104	0.9991	0.01–1	2.04	2.03	2.15
**14**	Y = 8 × 106x − 1.312 × 104	0.9991	0.01–1	2.68	2.22	2.81
**15**	Y = 3 × 106x − 2.803 × 104	0.9991	0.01–1	2.53	1.25	1.44
**16**	Y = 6 × 106x + 4.048 × 104	0.9992	0.01–1	1.68	2.79	2.85
**17**	Y = 2 × 106x − 1.297 × 103	0.9992	0.01–1	1.79	1.30	2.66
**18**	Y = 4 × 106x + 2.101 × 104	0.9990	0.01–1	2.05	2.74	1.57
**19**	Y = 6 × 106x + 5.441 × 104	0.9992	0.01–1	1.98	1.83	1.09

**Table 3:** Inhibitory activity (IC50, μM, 36 h) of compounds in G. *pentaphyllum* extracts in activated t-HSC/Cl-6 cells and LO2 cells.

Compounds	t-HSC/Cl-6	LO2	Compounds	t-HSC/Cl-6	LO2
**1**	>200	>200	**11**	43.6 ± 2.7 ***	>200
**2**	50.4 ± 1.4 ***	>200	**12**	>200	>200
**3**	>200	>200	**13**	105.6 ± 2.6 *	>200
**4**	126.4 ± 9.7 *	>200	**14**	>200	>200
**5**	>200	>200	**15**	>200	>200
**6**	99.9 ± 4.5 *	>200	**16**	44.7 ± 1.0 ***	>200
**7**	152.0 ± 7.4	>200	**17**	66.1 ± 3.8 ***	>200
**8**	68.8 ± 3.2 ***	>200	**18**	58.0 ± 2.9 ***	>200
**9**	113.6 ± 7.8 *	>200	**19**	28.1 ± 2.0 ***	>200
**10**	>200	>200	**Silymarin b**	185.3 ± 10.1	>200

The results were shown as the mean value ± SD. * *p* < 0.05, *** *p* < 0.001, when compared to LO2 values by Student’s t-test, Silymarin was used as positive control.

## Data Availability

Data available in a publicly accessible repository.

## Sample Availability

Samples of the compounds are not available from the authors.
